# Supplementary material for: The interactome of intact mitochondria by cross-linking mass spectrometry provides evidence for coexisting respiratory supercomplexes
Source: Mol Cell Proteomics. 2017 Dec 8;17(2):216–32. doi: 10.1074/mcp.RA117.000470 (PMC5795388; doi:10.1074/mcp.RA117.000470)
Supplement: Supplemental Data [file supp_RA117.000470_133922_0_supp_23978_fzffwf.zip › spectra_annotation/mito_spectra_annotation/158-1-7-1-12-1.pdf]

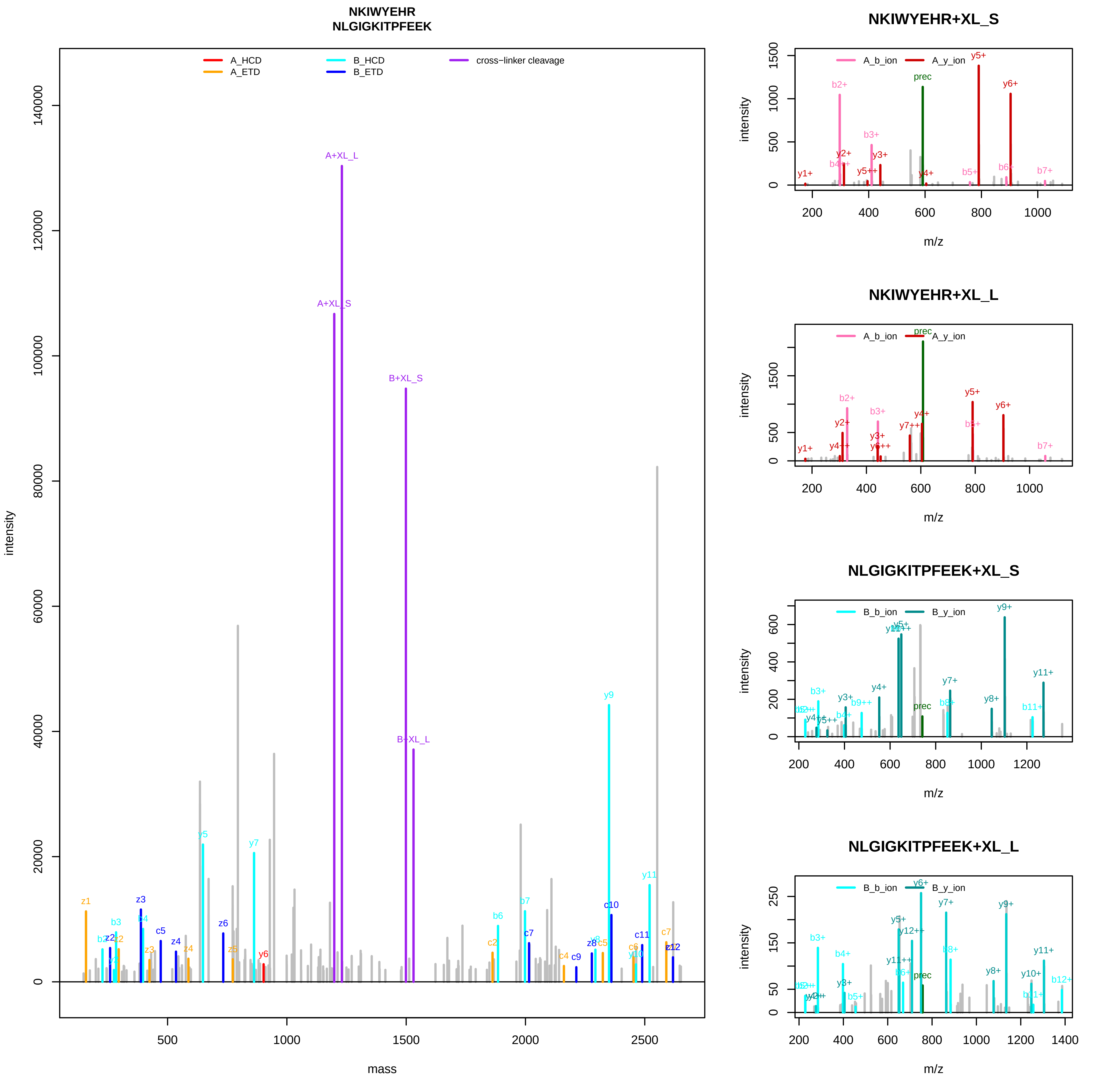
NKIWYEHR
NLGIGKITPFEEK
A_HCD
A_ETD
B_HCD
B_ETD
cross−linker cleavage
A+XL_L
A+XL_S
B+XL_S
B+XL_L
y9
y11
y5
y7
b6
b7
c10
c11
c7
z12
z1
z3
b3
b4
c5
z4
z6
y6
c2
c4
c9
z8
c5
c6
y10
intensity
mass
0
20000
40000
60000
80000
100000
120000
140000
500
1000
1500
2000
2500
NKIWYEHR+XL_S
A_b_ion
A_y_ion
prec
b2+
b3+
y2+
y3+
y5+
y6+
y1+
b5+
b7+
intensity
m/z
NKIWYEHR+XL_L
A_b_ion
A_y_ion
prec
b2+
b3+
y2+
y5+
y6+
y4+
y1+
b7+
intensity
m/z
NLGIGKITPFEEK+XL_S
B_b_ion
B_y_ion
y9+
y11+
y7+
y8+
y4+
y3+
b3+
prec
intensity
m/z
NLGIGKITPFEEK+XL_L
B_b_ion
B_y_ion
y6+
y7+
y9+
y11+
y8+
y10+
b12+
b3+
b4+
y3+
prec
intensity
m/z
